# Influence of UHMWPE on the expansion characteristics of expansive soil: Experimental studies

**DOI:** 10.1371/journal.pone.0326123

**Published:** 2025-07-07

**Authors:** Yonggang Huang, Hongri Zhang, Rong Shen, Xianliang Tan, Xinzhong Wang, Yuexing Wu

**Affiliations:** 1 School of civil engineering, Hunan city University, Yiyang, Hunan, China; 2 Guangxi Transportation Science & Technology Group Co., Ltd, Nanning, Guangxi, China; 3 Shanghai Jiao Tong University, Shanghai, China,; 4 School of civil engineering, Changsha University of Science and Technology, Changsha, Hunan, China; Shandong University of Technology, CHINA

## Abstract

This paper investigates the effectiveness of using Ultra-High-Molecular-Weight Polyethylene (UHMWPE) Fiber to improve expansive soil. The test results indicate that the amount and length of UHMWPE significantly affect the swelling pressure and free swelling ratio of the reinforced soil. The swelling pressure of reinforced expansive soil exhibits a significant negative correlation with both the UHMWPE content (*p* < 0.05) and its length. The free swelling ratio of reinforced expansive soil was significantly negatively correlated with both the content (*p* < 0.05) and length of UHMWPE. When the UHMWPE content exceeds 0.3% the weight of the soil, there is no significant decrease in the swelling pressure of the reinforced soil (*p* = 0.052, close to the threshold of significance). When the length of UHMWPE is greater than 9 mm, there is no significant decrease in the free swelling ratio of the reinforced soil (*p* = 0.165). Thus, it is recommended to use 0.3% UHMWPE with a length of 9 mm for optimal results. Swelling pressure, free swelling ratio and fiber content follow a quadratic polynomial relationship. While, the relationship between swelling pressure and fiber length is linear. The relationship between free swelling ratio and fiber length is a power function.

## 1 Introduction

Expansive soil is an unsaturated soil made up of a large quantity of hydrophilic clay minerals [1–4]. Engineering issues due to the distinct swelling, shrinkage and cracking properties of expansive soils have resulted in widespread research to improve them [5].

In 1957, Henri Vidal introduced the concept of reinforced soil. Subsequently, by 1967, several key reinforced – soil structures were constructed and implemented in Europe. Since the 1970s, a variety of non-metallic reinforced soil structures, including geotextile reinforced soil, which originated in 1971, have been developed in Europe, the United States, and other countries. Reinforced soil technology has been in development for over 50 years. Currently, rigid materials and metal bolt reinforcement technology have reached a high level of maturity. In recent years, various reinforcement technologies have been developed, and fiber reinforcement improvement technology, particularly the use of flexible reinforcement treatment, has captured the attention of scholars [6–7].

Previous studies have demonstrated that incorporating fibers (e.g., natural hay, vetiver grass, and synthetic basalt) into expansive soil can effectively suppress its swelling potential by forming a three-dimensional reinforcement network. This network restricts particle displacement during water absorption [8–9]. For instance, Abd El [10] observed that increasing hay fiber content reduced expansion deformation by up to 40%, while shear strength improved proportionally. Similar trends were reported for vetiver grass [9] and basalt fibers [8], suggesting that fiber reinforcement enhances soil stability through both mechanical interlocking and interfacial friction. Hu et al. [11] conducted indoor experiments combined with numerical simulations to verify that incorporating PE fiber into expansive soil can decrease the swelling pressure and free swelling ratio and enhance the soil’s shrinkage properties. Lei and Ding [12] added hemp fiber to expansive soil and performed tests to measure soil expansion. According to the results, the inclusion of hemp fiber can mitigate the expansion of expansive soil while demonstrating an optimal reinforcement ratio. In a series of indoor tests, Zhang and Shi [13] incorporated two types of polypropylene fibers with varying contents into expansive soil. These findings indicate that adding fibers can minimise shrinkage in fiber soil, while effectively enhancing its compressive and shear strength. In their study, B.R. and Ravideep [14] investigated the effect of varying nylon fiber content and length on the expansion and consolidation properties of expansive soil samples, using one-dimensional consolidation tests and nylon fiber reinforced expansive soil samples. Beyond laboratory-scale improvements, such stabilization techniques hold significant potential for real-world engineering applications. A recent study by Saleh et al. [15] revealed that optimizing subgrade soil properties could reduce flexible pavement thickness by 25%−30%, directly translating to lower construction costs and extended service life. These findings indicate that the inclusion of nylon fibers enhances the secondary consolidation properties of expansive soil.

Despite these advancements, conventional natural and synthetic fibers face challenges in harsh environments. For example, nylon and polypropylene fibers may degrade under cyclic wet-dry conditions due to hydrolysis [14], while natural fibers (e.g., hemp) are susceptible to biological decay [12]. These limitations highlight the need for a reinforcement material with superior durability. Ultra-high-molecular-weight polyethylene (UHMWPE) fiber, recently emerging as a high-performance synthetic material, offers exceptional tensile modulus (>100 GPa) and chemical inertness [16]. Its resistance to corrosion and abrasion makes it particularly suitable for expansive soil applications, where prolonged exposure to moisture and ionic solutions is common.

Beyond its mechanical superiority, UHMWPE fiber exhibits negligible degradation in acidic or alkaline soils [17–19], ensuring long-term stability in geotechnical applications. However, its potential for expansive soil modification remains underexplored. This study systematically investigates the effect of UHMWPE fiber content (0.1%−0.4% by weight) on the swelling pressure and free swelling ratio of expansive soil. We hypothesize that UHMWPE fibers, through their high stiffness and interfacial bonding with clay particles, will mitigate swelling by constraining volumetric strain during water uptake. The findings aim to establish a quantitative relationship between fiber dosage and swelling suppression efficiency, providing a basis for field application.

## 2 Materials

**Fig 1:**
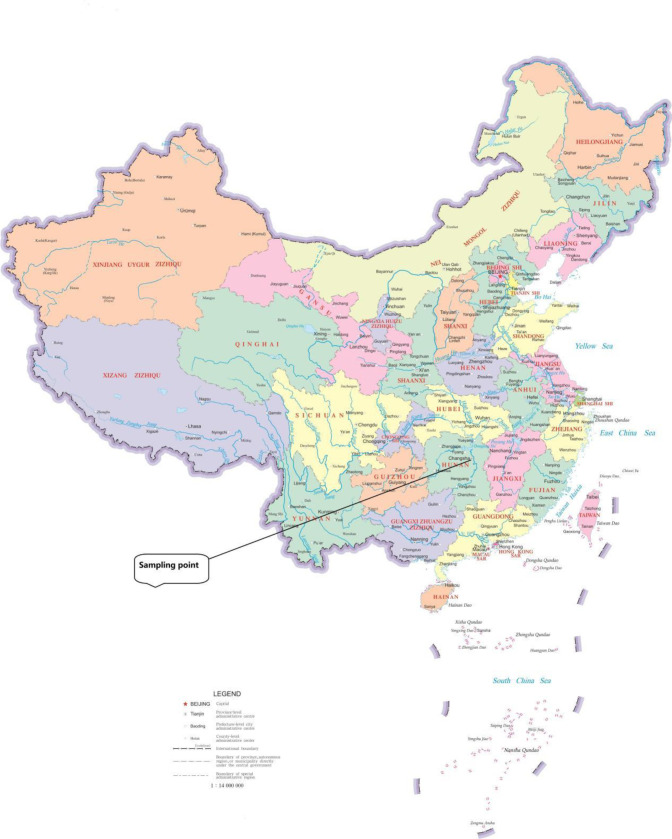
Sampling point (Source: http://bzdt.ch.mnr.gov.cn/. Map-examination No.:GS(2022)4308).

**Fig 2:**
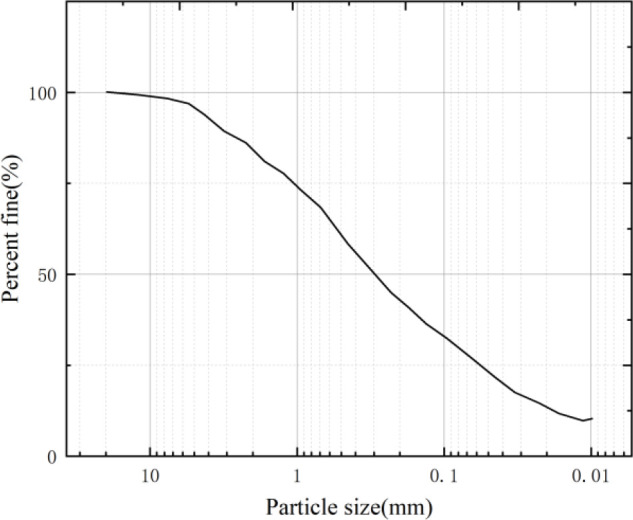
Soil partice-size distribution.

The soil sample was taken from an excavated slope of Shuxiang Road (112^o^58’28.06’‘N, 28^o^6’31.04“E) in Changsha (Fig 1). The soil samples were collected from a depth of 0.5–1.5 meters below the ground surface, and the soil layer showed obvious stratification characteristics, with a relatively uniform distribution of particle sizes within the sampling range. Fig 2 shows the aggregate grading curves of the soils used in the experiments. When the soil samples were collected, they were air dried, crushed and passed through a 2 mm sieve. After air drying, the moisture content was determined to be 4% according to the *Geotechnical Test Procedure* (SL237–0031999) [20]. At the same time, according to the *Geotechnical Test Procedure* (SL237–0111999, SL237–0071999, SL237–0241999) [21–23] to determine the free swelling ratio, maximum dry density, optimum water content, liquid limit water content, plastic limit water content, to obtain the basic parameters of the soil sample as shown in Table 1. The parameters characterizing the soil under investigation are as follows: the non – loading free swelling ratio is determined to be 50.0%, the optimum moisture content is measured as 20.0%, the plastic limit is found to be 20.0%, the liquid limit is established at 53.5%, and the maximum dry density is ascertained as 1.564 g/cm³.

**Table 1:** Test soil parameters.

Index	Non-loading free swelling ratio (%)	Optimum moisture content (%)	Plastic limit/%	liquid limit (%)	Maximum dry density (g/cm^3^)
Value	50.0	20.0	20.0	53.5	1.564

Table 2 shows the fundamental physical parameters of UHMWPF fiber. The parameters of the material are as follows: density is 0.97 g·cm ⁻ ³, diameter is 0.05 mm, tensile strength reaches 3000 MPa, elastic modulus is 90 GPa, and extension ratio is 3.5%. Soil samples were made by mixing soil with fibers of different lengths and quantities. Based on a range of values used in similar studies and our own pilot experiments, the fiber lengths are 6 mm, 9 mm, and 12 mm, respectively. The corresponding fiber contents are 0.1%, 0.2%, 0.3%, and 0.4%.

**Table 2:** UHMWPF parameters.

Parameter	Density (g^·cm-3^)	Diameter (mm)	Tensile strength (MPa)	Elastic modules (GPa)	Extension ratio (%)
Value	0.97	0.05	3000	90	3.5

## 3 Methods

In strict accordance with the *Standard for Soil Test Methods* (GBT 50123–2019) [24], the experimental procedures were meticulously executed. Initially, the soil samples underwent a series of pretreatment processes (No permits were required for the research site of this study. The site is a publicly accessible area where no special authorization is needed for conducting academic research activities. All research operations were carried out in compliance with public access regulations and did not involve any restricted or private areas.). These samples were air – dried, crushed, and subsequently sieved through a 2 – mm sieve to ensure uniform particle size distribution. Using an electronic balance with an accuracy of 0.01 g, a precise amount of the sieved soil was weighed. Based on the dry weight of the soil and the targeted water content of 21.3%, the quantity of distilled water required was calculated according to the formula:


$$M_{w}=Ms\times \left(w-w_{0}\right)/\left(1-w\right)$$ (1)

Where *M*_*w*_ is the mass of added water, *Ms* is the mass of dry soil, *w* is the target water content, and *w*_*0*_ is the initial water content of the sieved soil. The calculated amount of distilled water was then carefully measured and gradually added to the soil. To ensure homogeneous moisture distribution, the mixture was stirred thoroughly using a mechanical stirrer at a moderate speed for a duration sufficient to achieve complete wetting of all soil particles.

Subsequently, the treated soil samples were formed into standard ring – knife specimens. A specialized ring – knife pressing apparatus was employed to compress the soil into ring – knives with a diameter of 61.8 mm and a height of 20 mm. During the compaction process, a pressure sensor was integrated with the pressing device to monitor the applied pressure in real – time. By precisely controlling the pressure output of the pressing equipment, the compaction degree of the samples was accurately adjusted to reach 90%, in strict compliance with the relevant standards. This ensured the consistency and comparability of the ring – knife specimens for subsequent tests.

For the investigation of the effects of different fiber contents and lengths on the properties of expansive soil, various combinations were prepared. The fiber lengths considered were 6 mm, 9 mm, and 12 mm, while the fiber contents were set at 0.1%, 0.2%, 0.3%, and 0.4%. In the sample preparation stage, the soil was first placed in a clean and dry mixing vessel. Using an electronic balance with an accuracy of 0.1 mg, the appropriate mass of UHMWPE was accurately weighed according to the predetermined ratios. To achieve uniform fiber dispersion within the soil matrix, a two – step mixing approach was adopted. First, mechanical stirring at a low speed was performed for 3 minutes to initially distribute the fibers. Subsequently, manual stirring was carried out for an additional 5 minutes to ensure that the fibers were evenly dispersed throughout the soil, minimizing the occurrence of fiber agglomeration. This meticulous mixing process was crucial for obtaining samples with consistent fiber – soil interactions and reliable experimental results.

After the successful preparation of the samples, the free swelling ratio test was conducted [25]. The prepared ring – knife specimens with different fiber contents and lengths were carefully placed into a DGY - ZH0.8C bearing – type single – lever consolidation instrument. In accordance with the standardized procedures outlined in the *Standard for Soil Test Methods* (GBT 50123–2019) [24], an automatic water – injection apparatus was utilized to introduce distilled water with a temperature maintained at 20 ± 2°C into the consolidation instrument. This ensured that the samples were fully immersed in water under controlled conditions. During the test, a high – precision displacement sensor with an accuracy of 0.001 mm was employed to continuously monitor the vertical swelling displacement of the samples. Data were recorded at 15 – minute intervals until the samples reached a stable swelling ratio, which was defined as a condition where the displacement change in three consecutive measurements was less than 0.01 mm. The free swelling ratio of each sample was then calculated based on the measured swelling displacement using the formula:


$$FSR=(\left(h_{s}-h_{0}\right)/h_{0})\times 100\;\%$$ (2)

Where *FSR* is the free swelling ratio,*h*_*s*_ is the final height of the swollen sample, and *h*_*0*_ is the initial height of the sample.

For the swelling pressure test, the experimental setup was adjusted in accordance with the requirements of the *Standard for Soil Test Methods* (GBT 50123–2019) [24]. The hanging plate in the free swelling ratio test apparatus was replaced with a sand – filled bucket, and iron sand was used instead of conventional weights. After placing the sample in the consolidation instrument, the iron sand was added in a step – by – step loading manner, with an interval of 10 minutes between each loading increment. An electronic balance with an accuracy of 0.1 g was used to precisely measure the mass of the added iron sand. Throughout the loading process, the deformation of the sample was closely monitored. The swelling pressure was determined when the sample reached a stable deformation state, characterized by a deformation of less than 0.01 mm within 1 hour. The swelling pressure was calculated based on the mass of the added iron sand and the cross – sectional area of the sample using the formula:


P=(m×g)/A (3)

Where *P* is the swelling pressure, *m* is the mass of the added iron sand, *g* is the acceleration due to gravity, and *A* is the cross – sectional area of the sample.

To ensure the reliability and accuracy of the experimental data, a total of 5 parallel samples were prepared for each test condition. In the data – processing stage, Excel 2019 and SPSS 22.0 software were utilized for comprehensive data analysis. Excel 2019 was employed for data organization, basic statistical calculations, and the generation of preliminary data visualizations, such as scatter plots and line graphs, to provide an intuitive understanding of the data trends. SPSS 22.0 software was then used for more in – depth statistical analysis. Specifically, curve fitting was performed to determine the relationships between the addition amount and length of UHMWPE fibers and the swelling characteristics of expansive soil. The optimal fitting function forms were identified through a series of statistical tests. Additionally, a one – way analysis of variance (ANOVA) was conducted using the Pearson correlation coefficient to assess the significance of the relationships [26–27]. This statistical approach allowed for a quantitative evaluation of the influence of different factors on the experimental results, providing a solid basis for drawing scientific conclusions.


$$r=\sum \left[\left(x_{i}-\overset{―}{x}\right)\left(y_{i}-\overset{―}{y}\right)\right]/\sum \left[{\left(x_{i}-\overset{―}{x}\right)}^{2}*{\left(y_{i}-\overset{―}{y}\right)}^{2}\right]$$ (4)

Where *r* is Pearson’s correlation coefficient. *x*_i_ and *y*_i_ respectively represent each observation value in the two variables. $\overset{―}{x}$ and $\overset{―}{y}$ represent the average of two variables respectively.


df=n−k (5)

Where df is degrees of freedom. The *n* is the number of samples. The *k* is the number of conditions or variables that are restricted.


F=(Inter−groupvariance)/(Intra−groupvariance) (6)

If the *F* value is large, it means that the difference between the treatment groups is significant; if the *F* value is small, it means that the difference between the treatment groups is not significant.

*P* value is a parameter used to determine the results of hypothesis testing. The smaller the *P* value, the more significant the result is. In this paper, the threshold for *P* is 0.05.

## 4 Results

### 4.1 Influence of UHMWPE content on swelling pressure

The test results of swelling pressure of reinforced expansive soil are shown in the Table 3.

**Table 3:** The test results of swelling pressure of reinforced expansive soil.

Length of UHMWPE (mm)	UHMWPE content (%)				
	0.0	0.1	0.2	0.3	0.4
6	30.84	24.6	22.63	18.16	18.55
9	30.84	23.78	21.56	17.31	17.9
12	30.84	22.90	20.50	16.85	16.99

**Fig 3:**
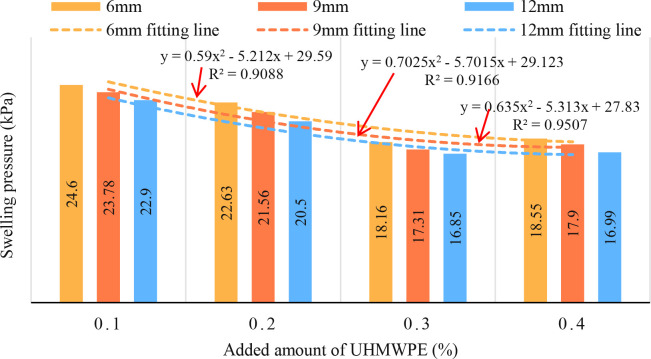
Influence of UHMWPE content on swelling pressure.

Table 3 shows that the addition amount (*F* = 780.32, *df* = 3, *p* = 0.000) and length (*F* = 78.660, *df* = 2, *p* = 0.000) of UHMWPE have a significant effect on the swelling pressure of reinforced expansive soil. The swelling pressure of reinforced expansive soil decreased with the increase of UHMWPE addition (*r* = −0.93, *df* = 9, *p* = 0.000). Significant differences were observed in expansive soil with varying UHMWPE additions, except for the 0.3% and 0.4% groups (*p* = 0.052). The swelling pressure of reinforced expansive soil does not decrease significantly when the content of UHMWPE exceeds 0.3%, there is no significant decrease in the swelling pressure of the reinforced soil (*p* = 0.052). Thus, 0.3% is the optimal addition amount (Fig 3).

The relationship between the swelling pressure and the fiber content accords with the law of quadratic polynomial development, and the swelling pressure decreases with the increase of the fiber content. The fitting coefficient of the fitting equation is above 0.9088. The fitting coefficients of 6 mm, 9 mm and 12 mm fiber reinforcement were 0.9088, 0.9166 and 0.9507, respectively. When the fiber content increases from 0.2% to 0.3%, the swelling pressures of fiber – reinforced soil with fiber lengths of 6 mm, 9 mm, and 12 mm decrease by 19.75%, 19.72%, and 17.8% respectively. When the fiber content increases from 0.3% to 0.4%, the swelling pressures of fiber – reinforced soil with fiber lengths of 9 mm and 12 mm also decrease by 3.3% and 0.82% respectively. The results showed that when the fiber content exceeded the critical value (0.3%), the fiber agglomeration effect weakened the improvement effect.

### 4.2 Influence of UHMWPE length on swelling pressure

**Fig 4:**
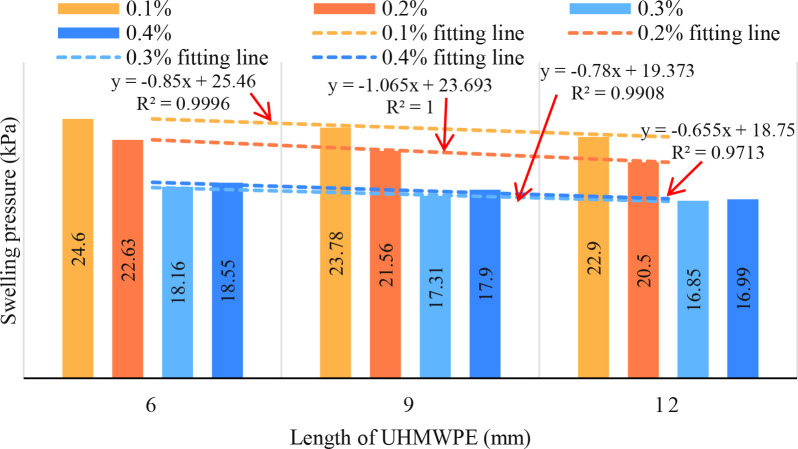
Influence of UHMWPE length on swelling pressure.

There was a negative correlation (*r* = −0.57, *df* = 9, *p* = 0.064) between UHMWPE length and the swelling pressure of reinforced expansive soil, with significant differences observed in expansive soil samples with different UHMWPE lengths. Fig 4 suggests that an increase in UHMWPE length can decrease the swelling pressure of expansive soil.

The relationship between swelling pressure and fiber length is linear function. The swelling pressure decreases linearly with the increase of fiber length. The swelling pressure decreases linearly with the increase of fiber length. The fitting coefficient of the equation is above 0.9713. The fitting coefficients of 0.1%, 0.2%, 0.3% and 0.4% fiber reinforcement were 0.9996, 1, 0.9908 and 0.9713, respectively. The results show that the swelling pressure decreases by more than 0.655 kPa with the increase of 1 mm fiber length.

### 4.3 Influence of UHMWPE content on free swelling ratio

**Fig 5:**
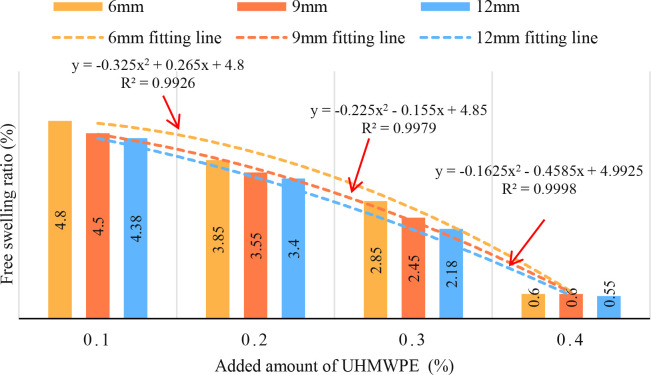
Influence of UHMWPE content on free swelling ratio.

The addition amount (*F* = 502.021, *df* = 3, *p* = 0.000) and length (*F* = 9.272, *df* = 2, *p* = 0.015) of UHMWPE have significant effects on the free swelling ratio of reinforced expansive soil. Significant difference were found in the free swelling ratio of the reinforced soil with varying UHMWPE content (*p* < 0.05). Additionally, the free swelling ratio of expansive soil decreased with the increase of UHMWPE content (*r* = −0.984, *df* = 9, *p* = 0.000) (Fig 5).

The relationship between free swelling ratio and fiber content conforms to the development law of quadratic polynomial function. With the increase of fiber content, the swelling pressure decreases. The fitting coefficient of the equation is above 0.9926. The fitting coefficients of 6 mm, 9 mm and 12 mm fiber reinforcement were 0.9926, 0.9979 and 0.9998, respectively. Taking 6 mm fiber as an example, when the fiber content increased from 0.3% to 0.4%, the free swelling ratio decreased by only 0.8% (from 16.85% to 16.99%), further verifying the negative impact of fiber agglomeration.

### 4.4 Influence of UHMWPE length on free swelling ratio

**Fig 6:**
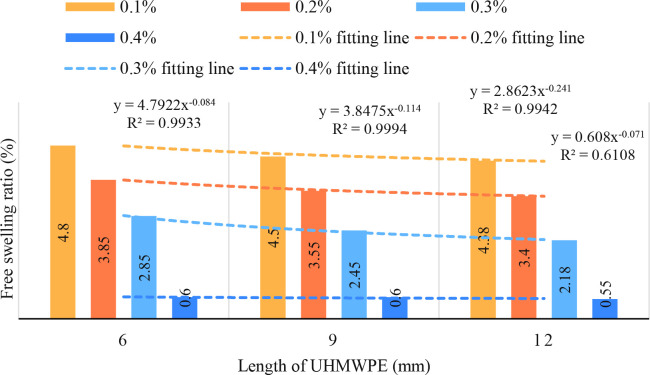
Influence of UHMWPE length on free swelling ratio.

Significant difference were not found in the free swelling ratio of reinforced soil with varying UHMWPE lengths (*p* = 0.165). The free swelling ratio of expansive soil decreased with the increase of length (*r* = −0.520, *df* = 9, *p* = 0.101). When the UHMWPE length exceeded 9 mm, there is no significant decrease in the free swelling ratio of the reinforced soil (*p* = 0.165), thus the optimal length of UHMWPE is 9 mm (Fig 6).

The relationship between free swelling ratio and fiber length accords with the law of functional development. With the increase of fiber content, the swelling pressure decreases. The fitting coefficient of the equation is above 0.6108. The fitting coefficients of 0.1%, 0.2%, 0.3% and 0.4% fiber reinforcement were 0.9933, 0.9994, 0.9942 and 0.6108, respectively. From 6 mm to 9 mm, the free swelling ratio decreased by 8.33%, 7.79%, 14.04% and 0%, respectively. From 9 mm to 12 mm, the free swelling ratio decreased by 2.67%, 4.23%, 11.02% and 8.33%, respectively. It shows that the marginal benefit of overlong fiber to restrain expansion is decreasing.

## 5 Discussions

The inclusion of fiber affects the swelling pressure and free swelling ratio of expansive soil. Incorporating fiber reduces the swelling pressure and free swelling ratio of expansive soil. Expansive soil’s swelling pressure and free swelling ratio reduce as the fiber’s content and length increase. If the addition amount exceeds 0.3%, there is no significant decrease in the swelling pressure.

**Fig 7:**
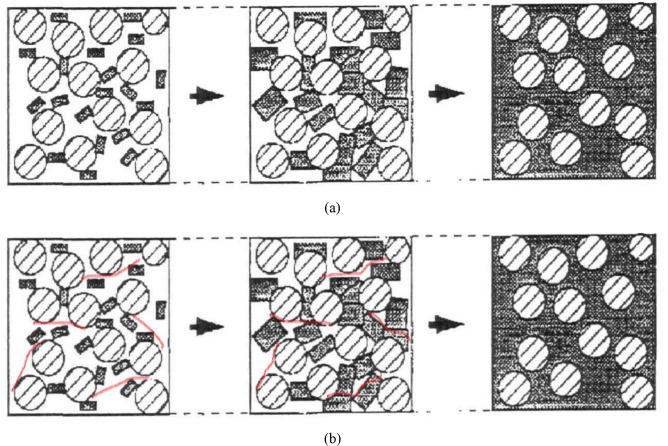
Shematic representation for the mechanism of the swelling pressure: (a)soil without added fiber; (b)soil with added fiber.

The phenomenon mentioned above is mainly due to the reinforcement effect of polypropylene fiber. This fiber hinders or delays the shear failure of the soil through its excellent tensile and pull-out properties [28]. The main reason for this phenomenon is the presence of friction and adhesion between soil particles and fibers. When soil expands, the montmorillonite mineral particles within it absorb water, causing their volume to increase. Initially, the surrounding pore space is filled. Once the montmorillonite particles have completely filled the pore space, they become restricted by the surrounding particles and cannot expand any further. The particles that are restricted generate an expansion force that is transmitted to the surrounding particles, resulting in a swelling pressure [29]. When fibers are added, the montmorillonite expansion resists not only the cohesion between the soil particles (Fig 7a), but also the friction and bonding between the soil particles and the fibers (Fig 7b). As a result, the expansion force transmitted between particles is reduced, and the macroscopically expressed swelling pressure is reduced.

However, a large amount of fiber could easily clump together in the soil. This manifests more obviously as the fiber content increases. Since the soil lacks the wrapping effect, the clumped fibers cannot exert their tensile properties fully. This causes a decrease in cohesion and a slow decrease in swelling pressure. Hence, increasing the amount of fiber content does not necessarily lead to better results.

If the fiber length more than 9 mm, the reduction in free swelling ratio is insignificant. This mainly occurs because, at the same fiber content, an increase in fiber length leads to a decrease in fiber number and increases the likelihood of fiber dispersion in soil. As a result, the number of tightly-bound fibers to the soil increases. This, in turn, improves the pull-out resistance of the fiber as a whole. Moreover, a longer fiber length results in a greater contact area with the soil, stronger interlocking friction, and higher interface strength. Consequently, the soil can better utilize the tensile properties of the fiber during the shear process, limiting expansive soil expansion, and subsequently reducing the swelling pressure and free swelling ratio.

Short fibers create numerous small voids on the sample’s surface. Long fibers experience significant tensile deformation without breakage. This reveals that long fibers can fully utilize their tensile properties during shearing, effectively delaying or preventing soil shear failure. Improving the cohesion of the soil effectively restricts the expansion of expansive soil. Additionally, shorter fibers result in a higher fiber count and greater tendency of agglomeration in the soil. During the soil shear process, dense fibers create a certain amount of inter-fiber friction, which can enhance the soil’s frictional strength to some extent. However, this phenomenon holds true solely when the fiber content is high, as low fiber content leads to better fiber dispersion in the soil and less agglomeration.

**Fig 8:**
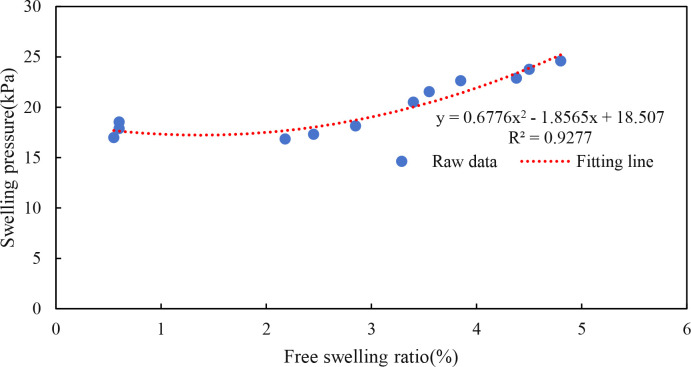
Swelling pressure versus free swelling ratio curve.

Meanwhile, as shown in Fig 8, the swelling pressure is positively correlated with the free swelling ratio, and the free swelling ratio increases with the swelling pressure. It shows that the swelling pressure or free swelling ratio can be derived by using the functional relationship approximation when the free swelling ratio or swelling pressure is known, which saves human and material resources. In addition, the fitting equation differs from the existing related literature mainly because of the structural variability of the soil, which indicates that the equation has limitations and the researcher needs to consider the specific engineering reality.

## 6 Conclusions

This research explored the efficacy of UHMWPE in improving expansive soil, with a focus on the impact of UHMWPE length and content on the soil’s swelling pressure and free swelling ratio. The following conclusions were drawn:

Influence Significance: The quantity and length of UHMWPE incorporated into expansive soil significantly affect its swelling pressure and free swelling ratio. This emphasizes the crucial role of UHMWPE in modifying the expansion – related properties of expansive soil, highlighting its potential as an effective soil – improvement material.

Correlation Patterns: A significant negative correlation exists between UHMWPE content and the swelling pressure of reinforced expansive soil (*p* < 0.05), and a similar trend is observed for the relationship between UHMWPE length and swelling pressure. The free swelling ratio of reinforced expansive soil also exhibits a significant negative correlation with both UHMWPE content (*p* < 0.05) and length. These correlations offer quantitative insights into the interaction mechanisms between UHMWPE and expansive soil, facilitating a deeper understanding of the soil – improvement process.

Optimal Parameters: When the UHMWPE content exceeds 0.3%, the reduction in swelling pressure becomes insignificant (*p* = 0.052). Similarly, when the UHMWPE length exceeds 9 mm, the decrease in the free swelling ratio is no longer significant (*p* = 0.165). Thus, an optimal UHMWPE content of 0.3% and a length of 9 mm are recommended. However, it’s essential to note that the determination of the optimal dosage should also consider its impact on other engineering properties, such as shear strength, to ensure comprehensive soil improvement.

Mathematical Relationships: The relationships among swelling pressure, free swelling ratio, and fiber content follow a quadratic polynomial model. The swelling pressure and free swelling ratio change non – linearly with increasing fiber content. The relationship between swelling pressure and fiber length is linear, with the swelling pressure decreasing linearly as the fiber length increases. The free swelling ratio and fiber length follow a power – function relationship. These mathematical relationships can be utilized to predict and simulate the behavior of UHMWPE – reinforced expansive soil, providing valuable tools for engineering design and theoretical research.

Inter – relationship between swelling parameters: The swelling pressure and free swelling ratio are positively correlated, following a quadratic polynomial development pattern. This relationship allows for the approximation of one parameter when the other is known, potentially reducing the need for extensive testing in certain engineering scenarios. However, due to the inherent variability in soil structure, the fitting equations have limitations. Practitioners should consider site – specific conditions when applying these relationships in real – world engineering projects.

In conclusion, this study provides valuable insights into the use of UHMWPE in improving expansive soil. The findings can serve as a reference for future research on soil improvement techniques and practical engineering applications involving expansive soil. Further research could explore the long – term performance of UHMWPE – reinforced expansive soil under different environmental conditions and its impact on other soil properties not fully investigated in this study.
